# Equity of child and adolescent treatment, continuity of care and mortality, according to age and gender among enrollees in a large HIV programme in Tanzania

**DOI:** 10.1002/jia2.25070

**Published:** 2018-02-27

**Authors:** Sumona Chaudhury, Ellen Hertzmark, Aisa Muya, David Sando, Nzovu Ulenga, Lameck Machumi, Donna Spiegelman, Wafaie W Fawzi

**Affiliations:** ^1^ Department of Epidemiology Harvard TH Chan School of Public Health Boston MA USA; ^2^ Department of Global Health and Population Harvard TH Chan School of Public Health Boston MA USA; ^3^ Management and Development for Health Dar es Salaam Tanzania

**Keywords:** gender, SDGs, equity, antiretrovirals, HIV‐infected, children, adolescents

## Abstract

**Introduction:**

Global scale up of anti‐retroviral therapy (ART) has led to expansion of HIV treatment and prevention across sub‐Saharan Africa. However, age and gender‐specific disparities persist leading to failures in fulfillment of Sustainability Development Goals, including SDG3 (achieving healthy lives and wellbeing for all, at all ages) and SDG5 (gender equality). We assessed ART initiation and adherence, loss to follow‐up, all‐cause death and early death, according to SDG3 and SDG5 indicators among a cohort of HIV‐infected children and adolescents enrolled in care in Dar‐es‐Salaam, Tanzania

**Methods:**

SDG3 indicators included young (<5 years) and older paediatric children (5 to <10 years), early adolescent (10 to <15 years) and late adolescent (15 to <20 years) age group divisions and the SDG5 indicator was gender. Associations of age group and gender with ART initiation, loss to follow‐up and all‐cause death, were analysed using Cox proportional hazards regression and with adherence, using generalized estimating equations (GEE) with the Poisson distribution. Associations of age group and gender with early death were analysed, using log‐Poisson regression with empirical variance.

**Results:**

A total of 18,315 enrollees with at least one clinic visit were included in this cohort study. Of these 7238 (40%) were young paediatric , 4169 (23%) older paediatric, 2922 (16%) early adolescent and 3986 (22%) late adolescent patients at enrolment. Just over half of paediatric and early adolescents and around four fifths of the late adolescents were female. Young paediatric patients were at greater risk of early death, being almost twice as likely to die within 90 days. Males were at greater risk of early death once initiated on ART (HR 1.35, 95% CI 1.09, 1.66)), while females in late adolescence were at greatest risk of late death (HR 2.44 [1.60, 3.74] <0.01). Late adolescents demonstrated greater non‐engagement in care (RR 1.21 (95% CI 1.16, 1.26)). Among both males and females, early paediatric and late adolescent groups experienced significantly greater loss to follow‐up.

**Conclusion:**

These findings highlight equity concerns critical to the fulfillment of SDG3 and SDG5 within services for children and adolescents living with HIV in sub‐Saharan Africa. Young paediatric and late adolescent age groups were at increased risk of late diagnosis, early death, delayed treatment initiation and loss of continuity of care. Males were more likely to die earlier. Special attention to SDG3 and SDG5 disparities for children and adolescents living with HIV will be critical for fulfillment of the 2030 SDG agenda.

## 1 Introduction

Global expansion of anti‐retroviral therapy (ART) has led to the marked success of prevention of mother‐to‐child transmission (PMTCT) programmes and increased enrolment into HIV care programmes across sub‐Saharan Africa 1. Despite significant strides in PMTCT globally, significant age and gender disparities exist in outcomes of efforts to prevent, diagnose, treat and manage HIV among children and adolescents, with significant implications for the fulfillment of SDG3 (achieving healthy lives and wellbeing for all, at all ages) and SDG5 (gender equality). Children are particularly vulnerable, experiencing a wide array of challenges, ranging from barriers to testing and treatment, to failures in continuity of care 1. Not all mothers receive antiretroviral medicines (ARVs), and delays in newborn testing and treatment persist with effects on perinatal transmission and disease management 2, 3. Global disparities in HIV‐related child mortality continue to disproportionately burden sub‐Saharan Africa 4. While global reductions in HIV‐related mortality have largely been driven by successful treatment of infections across other age groups, HIV‐related deaths among adolescents, particularly among girls, have risen during the same period 5, 6. Within the sub‐Saharan African HIV epidemic, adolescent girls are known to experience higher incidence of infections and barriers to care in late adolescence 7, while males experience relatively higher mortality 8, 9. Failure to address the age and gender‐specific needs of children and adolescents infected with HIV in sub‐Saharan Africa leads to failure to fulfill SDG3 and SDG5, with further interdependent failings in several Sustainability Development Goals (SDGs) 10.

The SDGs provide scope for the unification of development efforts across different sectors in the fulfillment of a single agenda for health 11. Arguably health is central to the fulfillment of the SDGs, being both as essential precursor and consequence of sustainable development 12. Integrating framework approaches can incorporate multiple SDG targets and provide opportunity for intersectoral and coordinated action for health 12. To achieve health for all children living with HIV in sub‐Saharan Africa, an integrated approach may strengthen links between targets within SDG3, such as SDG 3.3 (to end the AIDS and TB epidemics), 3.7 (sexual and reproductive health) and 3.8 (universal health coverage) and also strengthen links across different sectors. For instance, links between SDG3 and SD4 (education) targets, such as SDG 4.1 to 4.7, may emphasize relationships between child and adolescent sexual health and primary and secondary education. Links with SDG8 (economic productivity) targets 8.1 to 8.6 may facilitate measurement of the impacts of child and adolescent HIV on varying dimensions of the economic growth within the nation. An integrating framework approach can map differences in progress across all of the implicated sectors according to gender (to highlight progress towards SDG5) and according to international standards (to highlight progress towards SDG10 (reducing inequality within and among countries)) 8, 9, 10, 11, 12, 13, 14, 15, 16. Through mapping and measuring progress across the multiple sectors implicated by the HIV epidemic among children and adolescents, greater progress towards SDG17 may also be achieved, driving improvements in data required for greater accountability. Failure to achieve gender and age equity within the control of the African HIV epidemic, would lead to the failure of multiple interrelated SDG targets and objectives.

Shortfalls in approaches to HIV treatment and care and attendant age and gender‐specific disparities in treatment, health outcomes, and continuity of care within the health services of sub‐Saharan African countries have been increasingly well described over the past several years 8, 9, 13, 14, 15, 16, 17, 18, 19. This establishes a precedent for greater measurement of the impacts of these deficits on fulfillment of the SDGs as we move towards 2030. Tracking progress within an integrating SDG framework would allow for better capture of the interlinked impacts of child and adolescent health on varying aspects of sustainable development. Better monitoring and evaluation of progress towards 2030 goals additionally highlight novel intersectoral opportunities for intervention. Current efforts to track progress in delivering health for children and adolescents living with HIV largely rely on the UNAIDS 90‐90‐90 targets 20. Integrating UNAIDS 90‐90‐90 targets within an SDG framework would also shed light on opportunities to improve the health of children and adolescents living with HIV through intersectoral approaches 20.

Continued engagement with existing services is critical to the care of children living with HIV. Non‐engagement and loss to follow‐up are important indicators of failure in continuity of care 18, 19, 21. Although there is no singular gold standard measure to describe the varying forms of non‐engagement in care, non‐attendance has been described as a useful indicator of non‐engagement within this setting 19. Non‐engagement in care is associated with worse health outcomes among HIV‐infected patients 21. It is important to further understand the varying failures in the continuity of care of HIV‐positive children and adolescents enroled in services in high disease prevalence and impoverished settings 21. This prospective cohort study assesses early death and late all‐cause death, ART initiation, non‐attendance as a form of non‐engagement in care and loss to follow‐up. Utilizing SDG3 and SDG5 age and gender‐specific indicators, we sought to assess disparities in access to treatment, continuity of care and outcomes, among children and adolescents enrolled in a large sub‐Saharan African HIV care service in Tanzania.

## 2 Methods

### 2.1 Study population

The cohort included all HIV‐infected patients who were under 20 years of age at enrolment in services, between October 2004 and September 2014, within the urban HIV care and treatment clinics of Dar‐es‐Salaam, Tanzania (supported by Management and Development for Health (MDH) and the US President's Emergency Plan for AIDS relief (PEPFAR)). At the beginning of the study period the study catchment area included 28 clinics, which grew to 48 clinics by the end of the study period. Patients under the age of 15 years were considered paediatric for treatment purposes. The year of MDH enrolment was categorized, to reflect changes in Tanzanian National AIDS Control Program treatment guidelines, as 2004 to 2007, 2008 to 2011, and 2012 to 2014. Verbal consent for inclusion within research activities within the clinical programme was obtained from parents of children or from those patients over the age of 16 years, at enrolment, as appropriate. The Harvard TH Chan School of Public Health and Muhimbili University of Health and Allied Sciences Institutional Review Boards gave ethical clearance for this study (IRB 17‐1998).

### 2.2 Patient assessments, ART and follow‐up visits plan

All participants received free routine care and treatment for HIV according to Tanzanian National AIDS Control Program (NACP) Ministry of Health and Social Welfare guidelines, approved by the World Health Organization (WHO). Patients eligible for ART received free ART provision, subsidized by the Tanzanian government. Patients on ART were followed every 28 days for repeat physician assessment, refill of ART medication and counseling to discuss adherence, dosing and drug side effects. Late adolescent patients, who did not receive ART received supportive care and regular assessment of their eligibility for ART initiation every 6 months. Pediatric patients on care were reviewed monthly if under 5 years of age, or were reviewed every 3 months if above 5 years of age. ART initiation criteria differed by CD4 count percentage, age and period of enrolment. Before 2008, ART was initiated regardless of CD4 count percentage for children who were at WHO disease stage III/IV; for children with WHO stage I/II disease, ART was initiated if CD4 was below age‐adjusted thresholds (for those who were 12 to 18 months of age when CD4+ % was less than 25; between 19 and 59 months of age when CD4+ % was below 20; and for children 5 years of age and above when CD4+ % was below 15 (or CD4+ cell count <200 cells/ml)). For those <12 months of age, criteria changed in 2008. After 2008, ART was initiated if CD4+ % was <25 for those at WHO stage I/II and for all children who were <12 months of age, regardless of CD4+ % or WHO stage. Patients age 15 or older were treated by adult criteria. Weight and height were recorded at each visit. Data on ART and TB treatment, pregnancy and next appointment date were collected at each visit and laboratory measurements, and CD4 counts were performed and recorded every 6 months.

### 2.3 Definition of outcomes

Person‐time was divided between time “pre‐ART” and time “on ART”. Patients whose time of follow‐up coincided with their time to ART initiation were considered pre‐ART, as they had no follow‐up on ART. For both types of person‐time we considered 3 outcomes: “early death” (all cause death within 90 days of the beginning of that type of person‐time), ‘late death’ (all‐cause death more than 90 days after the beginning of that type of person‐time), “loss to follow‐up” (for pre‐ART patients, this means that the time to death or the study cutoff date was more than 180 days later than the scheduled appointment at their last recorded visit, or if there was no scheduled appointment recorded, more than 240 days later than their last visit). Patients on ART were considered lost to follow‐up, if time to death or the study cutoff date was more than 190 days after the scheduled appointment at their last recorded visit, or if there was no scheduled appointment recorded, more than 120 days later than their last visit. In addition, among patients on care, we considered the outcome of “ART initiation”. Among patients on ART, we considered the outcome of “non‐engagement in care”, defined as being 20% of the interval between visits later than the date of the scheduled appointment.

### 2.4 Data collection

Standardized forms were completed at each visit to detail clinical information. Standardized procedures for the measurement of height and weight were in place to record these at each clinic visit. Blood samples were taken at registration and every 6 months thereafter for hematologic, biochemical and immunologic profiling. Data assurance processes were in place at the point of entry including review checks, double data entry and supervisory checks for inconsistencies identified on second entry. Furthermore, weekly quality assurance checks of data were in place.

### 2.5 Statistical analysis

Primary determinants in the analysis included SDG3 and SDG5 indicators, generated through categorizing patients into five‐year age groups (0 to <5 young paediatric patients, 5 to <10 older paediatric patients, 10 to <15 early adolescent, 15 to <20 late adolescent) and according to gender, to compare intragroup differences in outcomes to assess equity. All analyses were undertaken separately among pre‐ART and on ART patients. Baseline characteristics were examined for the young and older paediatric, early and late adolescent age group, as well as gender groups. Means and standard deviations or medians with interquartile ranges were used to describe the centrality and distribution of continuous measurements and proportions to describe categorical measurements of baseline characteristics. Baseline covariates including WHO stage and immunodeficiency for age, HIV wasting syndrome (defined as at least 10% weight loss if the patient had diarrhoea and chronic weight loss and documented fever for at least 30 days not attributable to any concurrent condition), TB treatment, year of first clinic visit, district of clinic and marital and pregnancy status (for older adolescents). Immunodeficiency for age categories were constructed (with CD4 percent intervals cut at 25%, 30% and 35% for children <11 months of age, at 20%, 25% and 30% for children aged between 12 and 35 months and at 15%, 20% and 25% for children aged between 36 and 59 months with CD4 count intervals cut at 15% or 200, 350 and 500 cells/mm^3^ for children older than 5 years of age) in keeping with 2007 WHO guidelines 19. Age‐specific weight‐for‐length and BMI z‐scores were categorized at z‐scores of −3 and −2, categorized as severely malnourished (z‐score <−3), malnourished (z‐score ≥−3 by <−2), and not malnourished (z‐score ≥−2) (using weight‐for‐length z‐scores for children under 5 years of age and BMI z‐score for patients 5 years of age and over). The year of MDH enrolment was categorized, roughly coinciding with changes in the treatment guidelines of the Tanzanian National AIDS Control Program, as 2004 to 2007, 2008 to 2011, and 2012 to 2014. Missing indicators were created as necessary. For continuous variables the median value was imputed.

Crude rates and their 95% confidence intervals were determined using Poisson regression. Relative risks (RR) and 95% confidence intervals (CI) for early death were determined using generalized estimating equations (GEE) with the log link and the Poisson distribution to approximate log‐binomial regression 18. Associations of age group and gender with late death, ART initiation and loss‐to‐follow‐up were analysed, using Cox proportional hazards regression. Age group and gender associations with non‐attendance were examined with generalized estimating equations (GEE) with the log link and Poisson distribution for repeated measures of patient visits, using a compound symmetry working correlation and the robust variance.

Hazard ratios (HR) for receiving a prescription for ART were determined using proportional hazards models, adjusted for death and loss to follow‐up, using inverse probability weighting. Relative risks (RR) and 95% confidence intervals (CI) for non‐engagement in care were determined using GEE models with the log link, the Poisson distribution, and the exchangeable working covariance structure 22. For analyses using the subset of patients on ART, we also controlled for 4‐knot splines of time to ART initiation.

Given that the central exposures of interest in this study were baseline values (to include gender and age group at enrolment), we did not adjust for time‐varying covariates. We controlled for baseline covariates measured at enrolment to adjust for differences between age and gender groups. Covariates used included immunodeficiency for age and WHO HIV clinical stage based on 2007 WHO guidelines 19, presence of HIV wasting syndrome (as determined by the evaluating clinician), treatment for tuberculosis (TB), weight‐for‐height z‐scores, weight‐for‐length z‐scores for children under 5 and BMI z‐score for patients 5 and over), categorized as severely malnourished (z‐score <−3), malnourished (z‐score ≥−3 by <−2), and not malnourished (z‐score ≥−2) and district of Dar es Salaam. Potential confounders were selected, using *a priori* knowledge. Adjusted models included age group, gender, WHO HIV stage, BMI or weight‐for‐length z‐score (<−3, −3 to <−2, ≥−2), HIV wasting syndrome, immunodeficiency for age, TB treatment at enrolment, year of first clinic visit (2004 to 2007, 2008 to 2011, 2012 to 2014) and district of Dar es Salaam (Ilala, Kinondoni, Temeke). In all situations where the analysis used a subset of the data (e.g. late death, on ART), inverse probability weighting was used to adjust for censoring.

Cumulative incidence curves were constructed, using the Breslow estimator for multivariate proportional hazards models and adjusted for the following variables at enrolment: WHO HIV stage, immunodeficiency for age, HIV wasting syndrome, BMI or weight‐for‐length z‐score, pregnancy status, year of enrolment and district. Results were standardized to WHO HIV stage 1, CD4 high (500+ or 25%+), no HIV wasting syndrome, BMI or weight‐for‐length z‐score at least −2, not treated for TB, not pregnant, year 2004 to 2008, Ilala district.

For each outcome, univariate models of age group and gender SDG3 and SDG5 indicators, multivariate adjusted models, and models including interactions of age group with gender, were constructed. For proportional hazards models, *p*‐values for interaction were calculated, using the likelihood ratio test. For log‐Poisson models, *p*‐values for interaction were computed using the robust score test. All statistical tests were two‐sided, and *p*‐values of 0.05 or less were considered statistically significant. If the interactions were significant at the *p* = 0.05 level, we reported the results for age group within gender and for gender within age group. To test for non‐proportionality of hazards over time we divided the follow‐up time for each outcome at the mean and introduced two‐way interactions with age and gender. We selected backwards on these variables, retaining other model variables. No adjustment was made for multiple comparisons. Statistical analysis was undertaken using SAS 9.3 (Cary, North Carolina, USA).

## 3 Results

A total of 18,315 patients who were under 20 at MDH enrolment were included in our data. Of these, 10,790 (59%) were female, and 7325 (41%) were male. Among the paediatric and early adolescent patients there were approximately equal numbers of girls and boys, but among the late adolescents around 80% were young women. Around a third of the females who enrolled in late adolescence were pregnant at enrolment (Table 1).

**Table 1:** Basic characteristics of patients by age group and gender SDG3 and SDG5 indicators

		SDG3 indicators				SDG5 indicators	
	Total	Young paediatric	Older paediatric	Early adolescent	Late adolescent	Female	Male
Number of patients (N, (%))	18,315	7238 (40)	4169 (23)	2922 (16)	3986 (22)	10,790 (59)	7525 (41)
Age at enrolment (Mean (SD))	8.3 (6.5)	1.9 (1.5)	7.3 (1.4)	12.3 (1.4)	18.0 (1.4)	9.3 (6.8)	6.8 (5.6)
Follow‐up years (Median (IQR))	1.8 (0.4, 4.4)	1.5 (0.6, 3.0)	7.2 (6.1, 8.5)	12.2 (11.1, 13.5)	18.3 (17.0, 19.2)	1.2 (0.2, 3.9)	1.6 (0.2, 4.4)
Female (N, (%))	10,790 (59)	3, 781 (52)	2251 (54)	1614 (55)	3144 (79)	—	—
Married (N, (%))	1142 (6)	0 (0)	0 (0)	0 (0)	1142 (29)	1027 (10)	115 (2)
Pregnant female (N, (%))	1116 (6)	0 (0)	0 (0)	5 (0.2)	1111 (28)	1116 (10)	—
WHO stage (N, (%))							
I	4561 (31)	1925 (34)	669 (20)	437 (18)	1530 (48)	2963 (35)	1598 (25)
II	3556 (24)	1321 (23)	1067 (31)	619 (26)	549 (17)	1980 (23)	1576 (25)
III	5479 (37)	2012 (35)	1470 (43)	1109 (46)	888 (27)	2924 (35)	2555 (41)
IV	1142 (8)	424 (8)	220 (6)	248 (10)	250 (8)	580 (7)	562 (9)
Immune deficiency (N, (%))							
None	1665 (37)	954 (45)	4 (9)	83 (5)	624 (31)	897 (31)	768 (48)
Mild	975 (21)	392 (19)	6 (14)	89 (24)	411 (20)	657 (22)	318 (19)
Advanced	909 (20)	410 (20)	8 (19)	80 (21)	411 (20)	642 (22)	267 (17)
Severe	999 (22)	350 (16)	25 (58)	126 (33)	498 (25)	747 (25)	252 (16)
HIV wasting (N, (%))^a^	222 (1)	102 (2)	36 (1)	46 (2)	38 (1)	108 (1)	114 (2)
Weight for height z‐score (N, (%))							
<−3	4149 (23)	1757 (24)	965 (23)	619 (21)	808 (20)	2446 (23)	1703 (23)
−3 to <−2	1811 (10)	736 (10)	389 (9)	264 (9)	422 (11)	1051 (10)	760 (10)
−2+	12,337 (67)	4735 (66)	2811 (68)	2036 (70)	2755 (69)	7284 (59)	5053 (67)
On TB treatment (N, (%))	641 (4)	154 (2)	173 (5)	143 (6)	171 (5)	345 (4)	296 (5)
Only one visit (N, (%))	1854 (10)	794 (11)	337 (8)	213 (7)	510 (13)	1167 (11)	687 (9)
Year of 1st visit (N, (%))							
2004 to 2008	6732 (37)	3291 (45)	1724 (41)	1022 (35)	695 (17)	3674 (34)	3058 (41)
2009 to 2011	6962 (38)	2567 (36)	1593 (38)	1205 (41)	1597 (40)	4078 (38)	2884 (38)
2012 to 2014	4621 (25)	1380 (19)	852 (20)	695 (24)	1694 (43)	3038 (28)	1583 (21)
District of enrolment (N, (%))							
Ilala	7930 (44)	3089 (45)	1926 (46.7)	1332 (46)	1583 (40)	4460 (42)	3470 (47)
Kinondoni	5416 (30)	2015 (29)	1161 (28.2)	851 (29)	1389 (35)	3365 (32)	2051 (28)
Temeke	4544 (25)	1812 (26)	1038 (25.2)	713 (25)	981 (25)	2722 (26)	1822 (25)

a HIV wasting syndrome defined as at least 10% weight loss if diarrhoea, chronic weight loss, documented fever for 30 days not attributable to any condition other than HIV.

Median follow‐up time (IQR) was 656 (145, 1605) days. Among all patients, 12,299 (67%) received prescriptions for ART, including 658 (4% of those who received ART prescriptions) whose last follow‐up was the same as the date of the ART prescription (Table 1). Among pre‐ART patients, 331 (2%) had an early death (within 90 days of MDH enrolment), 197 a late death (3% of the 6315 who were followed for more than 90 days on care) and 5346 were lost to follow‐up. Among patients on ART, 365 (3%) had an early death), and 483 (5% of the 10,085 who were followed for more than 90 days after ART initiation) had a late death (Table 1 and Table 2). Among those on ART, the median time from MDH enrolment to ART initiation was 28 days (IQR (10, 137) days). Among pre‐ART patients, the hazard ratios for receiving a prescription for ART varied jointly by gender and age (gender‐age interaction *p* < 0.01), with early adolescents being most likely to receive prescriptions among the girls, but older paediatric patients being most likely to receive prescriptions among the boys (Table 3). Among late adolescents, males were much more likely to receive prescriptions than females (HR 1.32; 95% CI 1.21, 1.45). Among pre‐ART patients, follow‐up time was related to the effects of both age group and gender on receiving a prescription for ART. For both females and males, all age groups were less or approximately equally likely to receive ART prescriptions than older paediatric patients before the mean time, but much more likely to receive ART prescriptions after the mean time.

**Table 2:** Crude rates^a^ of early and late death and loss to follow‐up by age group and gender SDG3 and SDG5 indicators

SDG3 and SDG5 indicators	Early death		Late death		ART initiation		Loss to follow‐up	
	Years of follow‐up	Crude rate [95% CI]/100 person years	Years of follow‐up	Crude rate [95% CI]/100 person years	Years of follow‐up	Crude rate [95% CI]/100 person years	Years of follow‐up	Crude rate [95% CI]/100 person years
Patients pre‐ART								
Female	1327	12.2 [8.6, 17.4]	6038	1.7 [1.2, 2.6]	7293	96.2 [93.9, 98.4]	7363	47.5 [30.6, 73.7]
Young paediatric	2616	18.6 [11.7, 29.5]	2138	2.1 [1.2, 3.8]	2587	89.8 [86.2, 93.5]	2616	51.8 [26.5, 101.4]
Older paediatric	1673	8.8 [3.4, 22.7]	1391	1.8 [0.7, 4.5]	1658	95.0 [90.3, 99.7]	1673	34.9 [11.0, 110.9]
Early adolescent	954	13.2 [6.5, 26.6]	772	1.8 [0.6, 5.5]	944	130.1 [122.9, 137.4]	954	36.4 [9.6, 138.3]
Late adolescent	2120	6.2 [1.9, 20.1]	1736	1.2 [0.5, 2.7]	2103	89.6 [85.5, 93.6]	2120	57.0 [26.4, 123.4]
Male	4592	19.1 [13.6, 26.7]	3707	2.5 [1.5, 4.1]	4543	116.3 [113.2, 119.5]	4592	24.4 [24.4, 72.9]
Young paediatric	2070	24.2 [15.2, 38.3]	1649	3.0 [1.4, 6.4]	2041	107.3 [102.8, 111.8]	2070	54.5 [26.9, 110.2]
Older paediatric	1445	12.0 [5.3, 27.2]	1211	1.4 [0.6, 6.4]	1438	98.6 [93.5, 103.8]	1444	28.7 [7.9, 104.0]
Early adolescent	777	15.2 [7.6, 30.4]	626	2.6 [0.7, 9.2]	772	134.3 [126.2, 142.5]	777	28.5 [6.0, 135.4]
Late adolescent	300	20.4 [7.1, 58.4]	222	4.5 [2.4, 8.6]	292	218.8 [201.9, 235.8]	300	58.2 [12.3, 276.1]
Patients on ART								
Female	1497	11.6 [8.3, 16.3]	15,343	1.7 [1.0, 2.9]	—	—	16,480	17.3 [15.6 19.2]
Young paediatric	496	16.7 [10.6, 26.4]	5375	1.8 [0.7, 4.6]	—	—	5871	17.2 [14.1, 20.9]
Older paediatric	355	6.5 [2.0, 20.5]	6021	0.9 [0.4, 2.2]	—	—	4659	15.3 [12.4, 18.9]
Early adolescent	278	9.5 [5.6, 25.4]	3189	1.7 [0.4, 7.6]	—	—	3467	16.0 [13.2, 19.3]
Late adolescent	368	8.4 [4.4, 20.7]	2475	2.7 [1.1, 6.6]	—	—	2844	22.6 [18.4, 27.9]
Male	1161	16.4 [11.1, 24.3]	13,358	1.7 [1.0, 2.9]	—	—	14,519	16.6 [14.9, 18.6]
Young paediatric	474	19.4 [10.6, 35.5]	5362	1.5 [0.7, 3.1]	—	—	5836	17.0 [14.2, 20.4]
Older paediatric	322	10.3 [4.7, 22.4]	4033	1.4 [0.4, 4.2]	—	—	4355	15.8 [13.2, 19.0]
Early adolescent	232	15.5 [7.8, 30.8]	2832	2.1 [0.6, 7.4]	—	—	3064	15.7 [12.0, 20.5]
Late adolescent	133	22.5 [7.5, 68.1]	1131	2.7 [0.7, 10.8]	—	—	1264	20.2 [14.2, 28.9]

a Crude rates computed by Poisson regression. N.B. Person‐years may not add to totals because of rounding errors.

**Table 3:** Adjusted relative risks of early and late death, loss to follow‐up, ART initiation and late attendance among patients pre‐ART or on ART

Patients pre‐ART	SDG3 and SDG5 indicators	Early death RR [95% CI] *p*‐value	Late death HR [95% CI] *p*‐value	Loss to follow‐up HR [95% CI] *p*‐value	ART‐initiation RR [95% CI] *p*‐value
aNon‐proportionality test (*p*‐value)		—	0.25	0.63	<0.01
	Female Male	REF 1.24 [0.99, 1.54] 0.06	REF 1.01 [0.71, 1.42] 0.98	—	—
	Young paediatric Older paediatric Early adolescent Late adolescent	2.33 [1.71, 3.18] <0.01 REF 1.19 [0.80, 1.76] 0.39 1.13 [0.72, 1.77] 0.60	1.45 [0.93, 2.27] 0.10 REF 0.92 [0.52, 1.64] 0.79 0.78 [0.40, 1.50] 0.45	—	—
Gender–age interaction (*p*‐value)		0.91	0.30	<0.01	<0.01
Young paediatric	Female Male	—	—	REF 0.94 [0.87, 1.02] 0.16	REF 1.06 [1.00, 1.13] 0.04
Older paediatric	Female Male	—	—	REF 0.79 [0.70, 0.80] <0.01	REF 1.14 [1.06, 1.23] <0.01
Early adolescent	Female Male	—	—	REF 0.76 [0.64, 0.89] <0.01	REF 1.01 [0.93, 1.10] 0.29
Late adolescent	Female Male	—	—	REF 0.62 [0.53, 0.73] <0.01	REF 1.32 [1.21, 1.45] <0.01
Female	Young paediatric Older paediatric Early adolescent Late adolescent	—	—	1.35 [1.22, 1.49] <0.01 REF 0.89 [0.78, 1.01] 0.08 1.99 [1.79, 2.22] <0.01	1.01 [0.95, 1.08] 0.68 REF 1.19 [1.10, 1.28] <0.01 0.83 [0.77, 0.90] <0.01
Male	Young paediatric Older paediatric Early adolescent Late adolescent	—	—	1.27 [1.15, 1.41] <0.01 REF 0.85 [0.72, 1.00] 0.05 1.56 [1.30, 1.87] <0.01	1.08 [1.01, 1.15] 0.03 REF 1.06 [0.97, 1.14] 0.97 [0.88, 1.07] 0.49
**Patients on ART**	**SDG3 and SDG5 indicators**	**Early death** RR [95% CI]; *p*	**Late death** HR [95% CI]; *p*	**Loss to follow‐up** HR [95% CI]; *p*	**Non‐engagement in care** RR [95% CI]; *p*
aNon‐proportionality test (*p*‐value)		—	0.02	<0.01	—
	Female Male	REF 1.35 [1.09, 1.66] <0.01	—	REF 1.01 [0.96, 1.07] 0.76	REF 1.01 [0.98, 1.03] 0.66
	Young paediatric Older paediatric Early adolescent Late adolescent	1.96 [1.44, 2.68] <0.01 REF 1.47 [1.04, 2.09] 0.03 1.27 [0.87, 1.85] 0.22	—	0.93 [0.87, 1.00] 0.05 REF 0.95 [0.87, 1.03] 0.21 1.05 [0.95, 1.16] 0.31	1.06 [1.02, 1.09] <0.01 REF 1.12 [1.08, 1.16] <0.01 1.21 [1.16, 1.26] <0.01
**Patients on ART**	**SDG3 and SDG5 indicators**	**Early death** HR [95% CI]; *p*	**Late death** RR [95% CI]; *p*	**Loss to follow‐up** HR [95% CI]; *p*	**Non‐engagement in care** RR [95% CI]; *p*
Gender–age interaction (*p*‐value)		0.38	0.05	0.90	0.75
Young paediatric	Female Male	—	REF 0.86 [0.66, 1.13] 0.28	—	—
Older paediatric	Female Male	—	REF 1.61 [1.11, 2.36] 0.01	—	—
Early adolescent	Female Male	—	REF 1.29 [0.90, 1.83] 0.17	—	—
Late adolescent	Female Male	—	REF 0.86 [0.59, 1.26] 0.55	—	—
Female	Young paediatric Older paediatric Early adolescent Late adolescent	—	1.96 [1.33, 2.89] <0.01 REF 1.87 [1.23, 2.86] <0.01 2.44 [1.60, 3.74] <0.01	—	—
Male	Young paediatric Older paediatric Early adolescent Late adolescent	—	1.67 [1.12, 2.48] <0.01 REF 1.43 [0.98, 2.09] 0.06 1.30 [0.82, 2.08] 0.27	—	—

HR, Hazard ratio RR, Relative risk CI, confidence interval. For early death pre‐ART: Based on generalized estimating equations (GEE) models with the log link and the Poisson distribution, controlling for controlling for the year of MDH enrolment, presence of HIV wasting syndrome, immunodeficiency for age, WHO clinical HIV stage, TB treatment, weight‐for‐height category, district of Dar es Salaam, all at MDH enrolment. *p*‐value for interaction based on the robust score test. For early death on art: Based on generalized estimating equations (GEE) models with the log link and the Poisson distribution, controlling for controlling for the year of MDH enrolment, presence of HIV wasting syndrome, immunodeficiency for age, WHO clinical HIV stage, TB treatment, weight‐for‐height category, district of Dar es Salaam all at MDH enrolment; 4 knot splines of time to ART; weighted by the inverse probability of having follow‐up after ART initiation. *p*‐value for interaction based on the robust score test. For late death pre‐ART: Based on proportional hazards models controlling for the year of MDH enrolment, presence of HIV wasting syndrome, immunodeficiency for age, WHO clinical HIV stage, TB treatment, weight‐for‐height category, district of Dar es Salaam, all at MDH enrolment, weighted by the inverse probability of having more than 90 days of follow‐up. *p*‐value for interaction based on the likelihood ratio test. For late death on art: Based on proportional hazards models controlling for the year of MDH enrolment, presence of HIV wasting syndrome, immunodeficiency for age, WHO clinical HIV stage, TB treatment, weight‐for‐height category, district of Dar es Salaam, all at MDH enrolment; 4 knot splines of time to ART; weighted by the inverse probability of having more than 90 days of follow‐up on ART. *p*‐value for interaction based on the likelihood ratio test. For LTF pre‐ART: Based on proportional hazards models controlling for the year of MDH enrolment, presence of HIV wasting syndrome, immunodeficiency for age, WHO clinical HIV stage, TB treatment, weight‐for‐height category, district of Dar es Salaam, all at MDH enrolment. *p*‐value for interaction based on the likelihood ratio test. For LTF on ART: Based on proportional hazards models controlling for the year of MDH enrolment, presence of HIV wasting syndrome, immunodeficiency for age, WHO clinical HIV stage, TB treatment, weight‐for‐height category, district of Dar es Salaam, all at MDH enrolment; 4 knot splines of time to ART; weighted for inverse probability of ART initiation. *p*‐value for interaction based on the LRT. For ART initiation: Based on proportional hazards models controlling for the year of MDH enrolment, presence of HIV wasting syndrome, immunodeficiency for age, WHO clinical HIV stage, TB treatment, weight‐for‐height category, district of Dar es Salaam, all at MDH enrolment. The *p*‐value for interaction based on the likelihood ratio test. For poor engagement in care on ART: Based on generalized estimating equations (GEE) models with the log link and the Poisson distribution, controlling for controlling for the year of MDH enrolment, presence of HIV wasting syndrome, immunodeficiency for age, WHO clinical HIV stage, TB treatment, weight‐for‐height category, district of Dar es Salaam; 4 knot splines of time to ART; weighted by the inverse probability of having follow‐up after ART initiation. *p*‐value for interaction based on the robust score test.

a Non‐proportionality test based on age‐gender interaction over time (where the interaction was statistically significant at *p* < 0.05 level further segregation of results by time presented within supporting information).

SDG3 indicators demonstrated that among pre‐ART patients, young paediatric patients were most likely to experience both early and late death. Risk of early death was highest in pre‐ART patients but remained comparably high among patients on ART. Follow‐up time did not influence the hazard ratios for late death. SDG3 indicator for late adolescents was most strongly associated with loss to follow‐up, followed by the young paediatric group. Neither SDG3 nor SDG5 indicators were related to late death. SDG3 was implicated within loss to follow‐up, being greater among young paediatric patients, but higher still for both genders in late adolescence (Table 3).

Among patients on ART, both SDG3 and SDG5 indicators were implicated with males, young paediatric and early adolescent patients being more likely to experience early death. Young paediatric patients were the least likely to be lost to follow‐up. Follow‐up time did not influence the hazard ratios for these outcomes. Among patients on ART, follow‐up time affected the hazard ratios for age group for the late death outcome, with a halving of the hazard ratio for young paediatric patients relative to older paediatric patients in both genders after the mean follow‐up time. Both before and after the mean follow‐up time, hazard ratios for the other age groups relative to the older paediatric group were more extreme among females than among males.

SDG3 indicator for late adolescence was most significantly implicated with regards to poor engagement in care. For both genders late adolescents were more likely to be lost to follow up and non‐engaged in care. Among pre‐ART patients SDG 3 and SDG5 indicator interactions were significant for loss to follow‐up and ART initiation, but not among those patients already on ART for loss to follow up and non‐engagement.

**Figure 1:**
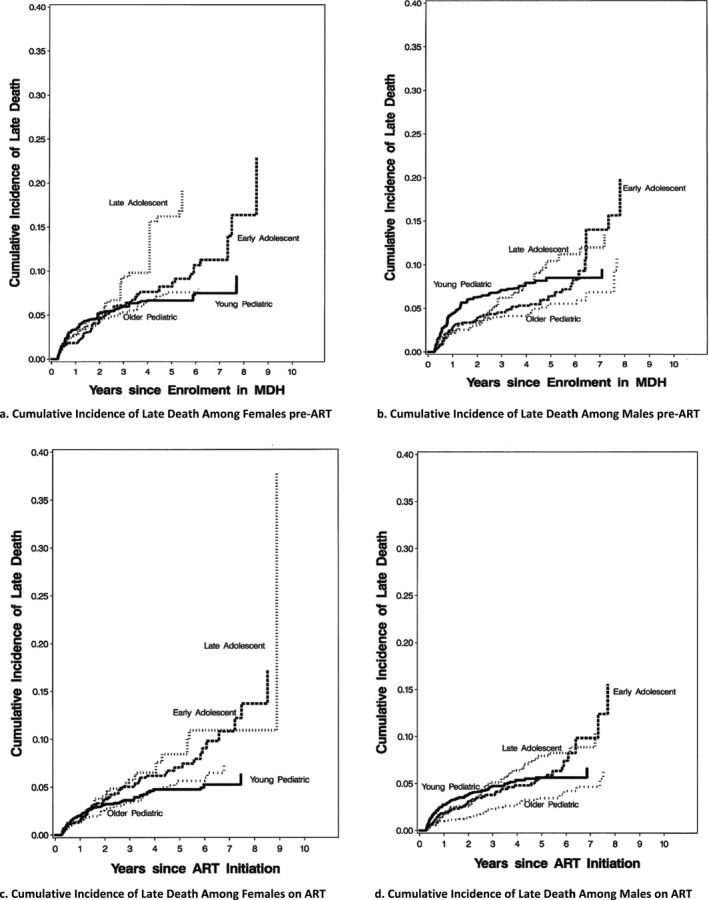
Adjusted cumulative incidence of death by Age Group and Gender SDG3 and SDG5 indicators. **(a)** Cumulative incidence of late death among females pre‐ART. **(b)** Cumulative incidence of late death among males pre‐ART. **(c)** Cumulative incidence of late death among females on ART. **(d)** Cumulative incidence of late death among males on ART

Figure 1 shows the cumulative incidence of death was generally more severe among males than females, with highest cumulative incidence of death being among early adolescent males. Early adolescents generally experienced the highest cumulative incidence of death followed by late adolescents, then paediatric patients (younger, followed by older paediatric patients, among females (see Figure 1a,c)). (Where there was suggestion of non‐proportionality and the further test for age‐time interaction was significant at the *p*‐value results were further disaggregated by time and presented in the supporting information).

**Figure 2:**
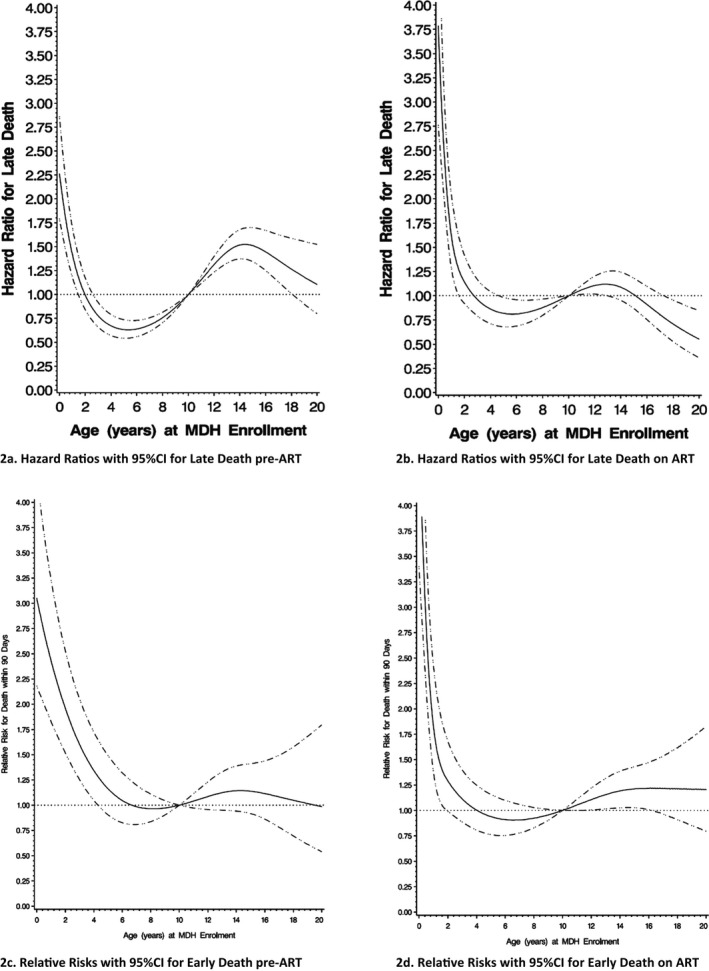
Hazard ratio for death according to age in years at enrolment (relative to rate at Age 10). **(a)** Hazard ratios with 95% CI for late death pre‐ART. **(b)** Hazard ratios with 95%CI for late death on ART. **(c)** Relative risks with 95% CI for early death pre‐ART. **(d)** Relative risks with 95%CI for early death on ART

Figure 2 shows that the overall hazard ratio for death according to age at enrollment, was highest shortly after birth, decreasing sharply until around age 6, and then increasing into late adolescence, when compared to death at aged 10 years at enrolment. Patients on ART generally experienced more rapid declines in relative risk of death, than patients on care, until around 5 years of age at MDH enrolment (see Figure 2c and 2d)).

## 4 Discussion

Current experience of enrollees in HIV care and treatment programmes across sub‐Saharan Africa is being increasingly well described 7, 8, 9, 10, 11, 12, 13, 14, 15, 16. Both female 3, 5, and male 7, 8, 9 children and adolescents living with HIV experience age and gender‐specific inequities in access to and continuity of HIV treatment and care with significant implications for the fulfillment of SDG3 and SDG5. Given the centrality of health for all ages and gender equality within the SDG agenda, intersectoral opportunities to protect and promote the health of children and adolescents living with HIV in sub‐Saharan Africa must be further examined.

It has been well recognized that young children living with HIV remain vulnerable to shortcomings in testing 2 and linkage to treatment 3, with potential consequence for their survival. Our findings corroborate that young paediatric patients remain particularly vulnerable to early death. Both in terms of SDG3 and SDG5 indicators, this study highlights that the young paediatric age group and male gender children are particularly at risk of early death, even when linked with care. This may be due to delays in testing or linkage to treatment once test results are available. Risk of early death was highest for the pre‐ART young children, but remained high among those on ART. But these findings suggest further measures must be taken to improve care during early life for these high‐risk groups.

Recent evidence also suggests that adolescents remain vulnerable to poor adherence and loss of retention within HIV care programmes 14, 15, 16. Our findings further confirm that the SDG3 indicator of late adolescence was particularly implicated in loss to follow up and poor engagement with care. SDG3 and SDG5 interactions being significant among the pre‐ART children and adolescents, implies significant age and gender differences in patterns of engagement with care, particularly prior to ART initiation. This further supports the assertion that fulfillment of SDG3 and SDG5 for children living with HIV prior to ART initiation depends on age and gender specific strategies to address engagement with care.

SDG 5 indicators were implicated significantly for early death in our findings. Additionally, crude death rates were the highest among males, being worst among the younger paediatric patients, reducing among the older paediatric patients, before worsening with increasing age category into late adolescence. Higher death rates among males may be explained by later presentation and greater non‐enegagement with care among 14, 23, 24. Adolescent males are known to access HIV services less frequently than females in sub‐Saharan Africa 9, 13. The greater majority of our adolescent patients were female with many engaging in care during pregnancy. This has been thought to be partially due to the successful expansion of national PMTCT programs, with adolescent females being supported and encouraged to access services and be tested for HIV during pregnancy; hence presenting at an earlier stage of disease. However, there may be more generalized barriers to access and adherence that specifically affect males, such as community stigmatization of health‐seeking behavior, reputational damage for those seeking HIV testing and treatment, social norms surrounding HIV status disclosure and clinic opening hours conflicting with hours of employment are reported by males in qualitative investigation 25, 26, 27, 28. Higher mortality among males who have never sought care has been more recently highlighted 9. Delays to presentation to services may significantly impact survival among males once treatment is started. Expansion of national programming to specifically address the intersection of SDG5 and SDG3 concerns, may seek to remove barriers for adolescent male attendance and retention within HIV care and treatment programmes and assist in altering community perception to achieve cultural normalization of male health‐seeking behavior among adolescents living with HIV 27.

Within the context of an integrating framework of SDGs, leveraging the educational sector could facilitate changes within primary and secondary education to improve adolescent male knowledge and attitudes towards health (SDG4.1‐4.7). Demonstrating links between the health of males of sexually active ages and consequent economic productivity, both through the education sector (SDG4.1‐4.7) and employment sector (SDG8.1‐8.6) could further highlight opportunities for intervention. Integrating SDG approaches may ultimately allow for greater alignment of intersectoral efforts in achieving the unifying objective of improved control of and response to the HIV epidemic for children and adolescents living with HIV.

Overall SDG3 and SDG5 indicator findings point to a number of key stages of life during which more rigorous attention to age‐ and gender‐specific needs may render significant benefits. Adolescents living with HIV remain particularly vulnerable to health inequity within HIV services, experiencing greater non‐adherence, loss to follow‐up and higher cumulative risk of death, than across other age categories. Those who enroled in care in early adolescence within our cohort had the highest cumulative mortality overall, across the age groups, with males having higher cumulative risk of death across every age group. Paediatric and early adolescent patients within our cohort may have been the most likely to have been perinatally infected, representing particularly high‐risk groups (although age at enrolment can offer only a crude indicator of timing of infection). Higher cumulative risk of death among those enrolled during early adolescence, may point to longer duration of perinatally acquired HIV infection and shortcomings in management of earlier infection. More rigorous attention to testing and linkage to care for children affected by HIV in early life, or loss of retention within care, during earlier childhood may reduce the impacts on mortality in early life 2, 3, 15, 16. Those enrolling in early adolescence may have experienced a lack of sustained management of their disease, or a repeated number of enrolments across different HIV services. Strengthening linkages for testing and treatment of infants once mothers are enrolled in PMTCT, and continued engagement with children once born within PMTCT programmes may diminish losses in continuity of care, that may be worsening the survival of perinatally infected children. The second highest cumulative risk of death, according to age group, was found among late adolescents, who may have been more likely to be sexually infected. Hence, later ART initiation, loss to follow‐up and worse non‐adherence in late adolescence, may lead to greater cumulative risk of death with age during adolescence. Hence, interventions supporting focused engagement, adherence and retention, within care, could save many years of life for adolescents living with HIV in sub‐Saharan Africa 15, 16. Strategies for improving services for adolescents have been described 27. Tanzania has made significant progress in incorporating evidence within national strategies for mapping HIV service delivery strategies for adolescents 27, 28. National efforts may be additionally better substantiated by incorporation of integrating SDG frameworks in the future.

SDG5 was implicated in our findings with females in late adolescence being more likely to experience delayed ART initiation. Many of the adolescent girls in our cohort were pregnant at enrolment, representing an important time during which adolescent girls access services. Adolescent females may generally present at an earlier disease stage during a high‐risk period of sexual reproductive life. However, a lack of integration of sexual and reproductive health services within HIV services (SDG 3.7) may lead to discrimination against women and girls in sub‐Saharan Africa 30, 31. Adolescent girls are vulnerable to sexually transmitted infections and require access to effective sexual, reproductive and HIV health services. High quality sexual and reproductive health services may mitigate incident HIV infections and unwanted pregnancies among adolescents (SDGs 3.3, 3.7, 3.8) 7. Failings in services for both adolescent males and females have important consequences for the fulfillment of SDG 3.8, limiting the achievement of effective universal health coverage, financial risk protection and access to quality essential health care services and medicines.

SDG3 indicators utilized in this analysis may have formed proxy indication of most likely mode of transmission, given that paediatric and early adolescent infections were more likely to have been perinatally transmitted and late adolescent infections were more likely to have been sexually transmitted. These limitations are usual to data from clinical cohorts, as engagement with clinical services relies on prevailing social and cultural norms concerning health‐seeking behaviors. Further measures for appraising SDG3, SDG5 and related SDG targets may be incorporated into future studies. Gains in health from application of integrating SDG framework approach to programme management, monitoring and evaluation could also be demonstrated in future research work. While studies from enrollees within HIV treatment services in sub‐Saharan African countries are becoming increasingly available, population‐based sampling is ideally needed for a more accurate estimation of population health 8, 9. Sampling only from sites of clinical care may lead to misclassification and measurement error 9, 31. For instance, those who die in childhood following maternal transmission of infection and those who are lost to follow‐up are unlikely to be reported or confirmed as dead. Limits to national systems of health‐related data collection limit the potential for fulfillment of SDG 17.18 (data monitoring and accountability) to track progress made in expanding treatment for HIV 15.

Despite the widespread expansion of ART treatment, further action is required to improve access to care and retention within health services 13, 14, 15, 16, 17. Additionally, emphasis is required on community and structural factors, which lead to poor health outcomes for this population, such as gender power inequity, stigma, poverty and lack of other resources needed to access care 32, 33, 34, 35. A holistic approach to the wellbeing of children and adolescents affected by HIV in sub‐Saharan Africa is needed to prevent failure of efforts to achieve the SDGs. Failings in fulfillment of SDG3 for HIV‐infected children and adolescents in sub‐Saharan Africa have implications for the fulfillment of several further SDGs, including SD4 (the commitment to inclusive and equitable quality education for all) and multiple aspects of SDG8 (sustained, inclusive and sustainable economic growth, full and productive employment and decent work for all). HIV‐infected adolescent males are experiencing higher cumulative risk of death and reductions in life expectancy 8, 9, preventing their primary and secondary education (impacting SDGs 4.1‐4.7) and contribution to the sustained economic growth of the nation (impacting SDGs 8.1.1, 8.2.1, 8.3.1, 8.5 and 8.6) 29. Well‐developed national commitments to the fulfillment of SDG 8b (to develop and operationalize a global strategy for youth employment) must recognize the impact of the HIV epidemic on adolescent lives. Strategies to improve adolescent engagement with and adherence and retention within HIV‐treatment programmes are necessary to mitigate the inter‐related failure of SDGs 3, 4 and 8 and the subsequent failure of SDG 10 (the reduction in inequality within and among countries).

Our data highlight important equity concerns for children and adolescents seeking HIV treatment and care services in sub‐Saharan Africa. Threats to the fulfillment of Sustainability Development Goals (SDGs) concerning health for all ages (SDG3), gender equality (SDG5), education (SDG4) and economic growth (SDG8) are highlighted by this study. Further downstream consequences of failed attempts to effectively treat and care for HIV‐infected children and adolescents within sub‐Saharan African health services would additionally undermine efforts to reduce inequalities within and among countries (SDG10). Efforts to improve national systems of data monitoring and accountability (SDG17) need to incorporate the measurement of the health of children and adolescents living with HIV, within integrating SDG frameworks in the future, to realize maximum benefits for population governance.

## 5 Conclusion

Strengthening health services for HIV‐infected children and adolescents in sub‐Saharan Africa will require special attention to age and gender inequities for fulfillment of the SDGs by 2030.

## Competing interests

The authors have no competing interests to declare.

## Authors' contributions

SC, EH, DS and WF designed the study. AM, DS, NU and LM contributed significantly to data collection. SC and EH performed the analysis. SC wrote the first draft.

All authors have read and approved the final manuscript.

## Supporting information


**Table S1.** Disaggregation of hazard ratios by age‐gender interactions over time for outcomes according to non proportionality tests
