# Supplementary material for: Equity of child and adolescent treatment, continuity of care and mortality, according to age and gender among enrollees in a large HIV programme in Tanzania
Source: J Int AIDS Soc. 2018 Feb 27;21(Suppl Suppl 1):e25070. doi: 10.1002/jia2.25070 (PMC5978660; doi:10.1002/jia2.25070)
Supplement: Supplementary file 1 — Table S1. Disaggregation of hazard ratios by age‐gender interactions over time for outcomes according to non proportionality tests [file JIA2-21-e25070-s001.docx]

| **Table 1. Disaggregation of Hazard Ratios by age-gender interactions over time for outcomes according to non proportionality tests** | | | | |
| --- | --- | --- | --- | --- |
| **Patients pre-ART** | **SDG3 and SDG5 indicators** | **Late death**  HR [95% CI] p-value | **Loss to follow-up**  HR [95% CI] p-value | **ART-initiation**  RR [95% CI] p-value |
| **Non-proportionality test (p-value)** | | 0.25 | 0.63 | <0.01 |
|  | **Female**  **Male** | --- | --- | --- |
|  | **Young pediatric**  **Older pediatric**  **Early adolescent**  **Late adolescent** | --- | --- | --- |
| **Gender–age interaction (p-value)** | | 0.30 | <0.01 | <0.01 |
| **Young pediatric**  **Before mean time** | **Female**  **Male** | --- | --- | REF  0.97 [0.91, 1.03); 0.34 |
| **Young pediatric**  **After mean time** | **Female**  **Male** | --- | --- | REF  1.62 [1.46, 1.78]; <0.01 |
| **Older pediatric**  **Before mean time** | **Female**  **Male** | --- | --- | REF  1.12 [1.04, 1.20]; <0.01 |
| **Older pediatric**  **After mean time** | **Female**  **Male** | --- | --- | REF  1.86 [1.66, 2.09]; <0.01 |
| **Early adolescent**  **Before mean time** | **Female**  **Male** | --- | --- | REF  0.92 [0.85, 1.01]; 0.07 |
| **Early adolescent**  **After mean time** | **Female**  **Male** | --- | --- | REF  1.54 [1.37, 1.73]; <0.01 |
| **Late adolescent**  **Before mean time** | **Female**  **Male** | --- | --- | REF  1.24 [1.13, 1.37]; <0.01 |
| **Late adolescent**  **After mean time** | **Female**  **Male** | --- | --- | REF  2.07 [1.84, 2.34]; <0.01 |
| **Female**  **Before mean time** | **Young pediatric**  **Older pediatric**  **Early adolescent**  **Late adolescent** | --- | --- | 0.87 [0.81, 0.93]; <0.01  REF  1.03 [0.95, 1.11]; 0.47  0.67 [0.62, 0.72]; <0.01 |
| **Female**  **After mean time** | **Young pediatric**  **Older pediatric**  **Early adolescent**  **Late adolescent** | --- | --- | 4.04 [3.58, 4.56]; < 0.01  REF  4.59 [3.98, 5.31]; < 0.01  4.79 [4.20, 5.48]; < 0.01 |
| **Male**  **Before mean time** | **Young pediatric**  **Older pediatric**  **Early adolescent**  **Late adolescent** | --- | --- | 0.76 [0.71, 0.81]; <0.01  REF  0.76 [0.71, 0.81]; <0.01  0.74 [0.67, 0.82]; <0.01 |
| **Male**  **After mean time** | **Young pediatric**  **Older pediatric**  **Early adolescent**  **Late adolescent** | --- | --- | 3.51 [3.10, 3.96]; <0.01  REF  3.80 [3.29, 4.39]; <0.01  5.34 [4.61, 6.18]; <0.01 |
| **Patients on ART** | **SDG3 and SDG5 indicators** | **Late death**  HR [95% CI]; p | **Loss to follow-up**  HR [95% CI]; p | --- |
| **Non-proportionality test (p-value)** | | 0.02 | <0.01 | --- |
|  | **Female**  **Male** | --- | REF  1.10 [0.95, 1.06]; 0.83 |  |
| **Before mean time** | **Young pediatric**  **Older pediatric**  **Early adolescent**  **Late adolescent** |  | 1.07 [0.97, 1.18]; 0.08  REF  0.90 [0.80, 1.01]; 0.08  1.29 [1.15, 1.45]; < 0.0 |  |
| **After mean time** | **Young pediatric**  **Older pediatric**  **Early adolescent**  **Late adolescent** |  | 0.85 [0.76, 0.94]; <0.01 REF  1.06 [0.93, 1.20]; 0.37  0.81 [0.69, 0.96)] 0.01 |  |
| **Patients on ART** | **SDG3 and SDG5 indicators** | **Late death**  RR [95% CI]; p | **Loss to follow-up**  HR [95% CI]; p | --- |
| **Gender–age interaction (p-value)** | | 0.05 | 0.90 |  |
| **Young pediatric** | **Female**  **Male** | REF  0.86 [0.66, 1.13] 0.28 | --- | --- |
| **Older pediatric** | **Female**  **Male** | REF  1.61 [1.11, 2.36] 0.01 | --- | --- |
| **Early adolescent** | **Female**  **Male** | REF  1.29 [0.90, 1.83] 0.17 | --- | --- |
| **Late adolescent** | **Female**  **Male** | REF  0.86 [0.59, 1.26] 0.55 | --- | --- |
| **Female**  **Before mean time** | **Young pediatric**  **Older pediatric**  **Early adolescent**  **Late adolescent** | 2.01 [1.40, 2.90]; <0.01  REF  1.85 [1.22, 2.79]; <0.01  2.42 [1.63, 3.62]; < 0.01 | --- | --- |
| **Female**  **After mean time** | **Young pediatric**  **Older pediatric**  **Early adolescent**  **Late adolescent** | 0.96 [0.52, 1.78]; 0.91  REF  1.97 [1.07, 3.66]; 0.03  2.38 [1.26, 4.53]; <0.01 | --- | --- |
| **Male before mean time** | **Young pediatric**  **Older pediatric**  **Early adolescent**  **Late adolescent** | 1.08 [0.76, 1.52]; 0.67  REF  1.47 [1.01, 2.15]; 0.05  1.30 [0.83, 2.02]; 0.25 | --- | --- |
| **Male after mean time** | **Young pediatric**  **Older pediatric**  **Early adolescent**  **Late adolescent** | 0.52 [0.28, 0.94]; 0.03  REF  1.57 [0.87, 2.85]; 0.14  1.27 [0.66, 2.47]; 0.48 | --- | --- |
